# Structural and Physical Properties of Alginate Pretreated by High-Pressure Homogenization

**DOI:** 10.3390/polym15153225

**Published:** 2023-07-28

**Authors:** Xiu Zhang, Jianrong Chen, Xuezhi Shao, Hongliang Li, Yongqiang Jiang, Yunkai Zhang, Dengfeng Yang

**Affiliations:** 1 College of Life Science and Technology, Guangxi University, Nanning 530004, China2114391078@st.gxu.edu.cn (X.S.); 2 Guangxi Key Laboratory of Marine Natural Products and Combinatorial Biosynthesis Chemistry, Guangxi Academy of Sciences, Nanning 530007, China; hongliang-li@gxas.cn; 3 Institute of Biology, Guangxi Academy of Sciences, Nanning 530007, China; 4 College of Food and Quality Engineering, Nanning University, Nanning 541699, China

**Keywords:** structural property, sodium alginate, extraction condition, high-pressure homogenization

## Abstract

To develop a high-efficient extraction method, we investigated the use of high-pressure homogenization (HPH) as a novel pretreatment technology for the extraction of sodium alginate (SA) from *Laminaria japonica*. After the single-factor experiment, the results demonstrated that under the conditions of 100 MPa HPH pressure, 4 cycles, pH 6.0, and 0.5% EDTA for 3.0 h, the optimized extraction yield of HPH reached 34%. To further clarify the effect on the structural properties of HPH-extracted SA, we conducted comprehensive analysis using SEM, FTIR, MRS, NMR, XRD, TGA, and a T-AOC assay. Our findings revealed that HPH pretreatment significantly disrupted the structure of *L. japonica* cells and reduced their crystallinity to 76.27%. Furthermore, the antioxidant activity of HPH-extracted SA reached 0.02942 mgVceq∙mg^−1^. Therefore, the HPH pretreatment method is a potential strategy for the extraction of alginate.

## 1. Introduction

Marine seaweed have gained a lot of attention as a third-generation renewable feedstock in recent years due to its high growth rate and biomass output, as well as its ability to be cultivated on a wide scale, without the need for arable land [1]. Furthermore, because brown macroalgae do not contain lignin, simple biorefinery processes such as milling, leaching, and extraction can separate the sugars for conversion into bioactive oligosaccharides, biofuels, and renewable chemicals. Brown algae is currently the main source of alginate, a linear acidic polysaccharide carbohydrate composed of β-D-mannuronic acid (M) and its C5 epimer α-L-guluronic acid (G) [2]. The performance of alginate is revealed to be strongly correlated with the M/G ratio [3,4]. Alginate mainly exists in various types of brown algae, with the molecular weight sequence being *Fucus vesiculosus* < *Ascophyllum nodosum* < *Sargassum fluitans* < *Laminaria japonica*. Among these seaweed species, *L. japonica* is currently the main source for alginate extraction due to its high content, large molecular weight, and ease of extraction [5].

Commercial production of alginate began in the late 1920s and eventually dominated the food ingredient market in the mid-20th century [6]. Large-scale industrial production commenced in the United States in 1929. In 1938, it gained approval from the Food and Drug Administration (FDA) for use in the food and pharmaceutical industries. Today, the market size of alginate is expected to reach USD 923.8 million by 2025 due to its increasing use in food and biomedical industries [7]. Alginate has numerous proven properties, such as being non-toxic, water-soluble, biodegradable, film-forming, gelling, thickening, anti-allergic, flocculating, chelating, and promoting plant growth [8,9,10,11,12,13,14]. At present, alginate has widespread application in various industries around the world. Apart from the previously mentioned food, medicine, printing, and dyeing industries, sodium alginate is also extensively used in water treatment and bioethanol production [15,16,17].

The extraction of alginate from seaweed is based on converting all the alginate to sodium salt and dissolving it in water, then removing impurities and recovering the alginate from the aqueous solution. There are two different methods for recovery. The first method involves adding acid to form insoluble alginate acid, which can be separated from the water. The second method involves adding calcium salt to form insoluble calcium alginate, which can also be separated [18]. The extraction of alginates from brown seaweed has been the subject of study for several decades in order to develop economic systems that can achieve high yields and control the molecular weight for various applications [19,20,21,22]. However, extraction methods using acid and alkaline can make the intermediate product alginate unstable and prone to degradation, and they also require multiple steps and are expensive [23]. Enzymatic hydrolysis, although a potential alternative, is currently hindered by high cost, high energy consumption, long extraction period and harsh conditions, and has not yet been successfully implemented in large-scale industrial production [24,25].

Traditional acid and aldehyde extraction methods are gradually being phased out due to environmental pressure. However, the extraction yield of sodium alginate is affected by the compact structure of seaweed cell walls, which makes them difficult to degrade. In order to improve the extraction yield, various pretreatment methods such as microwave-assisted extraction (MAE) [26], ultrasonic-assisted extraction (UAE) [27], complex enzyme hydrolysis (CE) [28], and complex enzyme–ultrasonic combined method (CE–UC) [29,30] have been introduced. These pretreatment methods are considered necessary and helpful in the extraction process. Therefore, selecting a suitable pretreatment method is a crucial step in the extraction process.

Alginate oligosaccharides (AOSs) obtained from the degradation of alginate have attracted significant attention due to their physiological characteristics, which include antioxidant, antimicrobial, anticancer, anti-inflammatory, immune regulation, and the promotion of plant growth [31,32,33,34]. Among the various methods used to produce AOS, the enzymatic method is considered the most promising due to its several advantages, such as higher specificity and efficiency, mild reaction conditions, and high reaction yields [35]. Alginate lyases (Alys) are enzymes that can cleave glycosidic bonds through a β-elimination reaction to depolymerize alginate into AOS. Sodium alginate, which has low crystallinity and large porosity, is more suitable as a substrate for the production of AOS.

HPH is a highly practical technology that is widely used in the pretreatment of various materials, including milk emulsion, lignocellulose pretreatment, and cell disruption [36,37,38,39]. However, to date, this technique has not been applied to extract SA from *L. japonica* cells. Annually, approximately 23 kt of alginate, 7.5 kt of agar, and 28 kt of carrageenan are extracted from 1000 kt wet seaweed [40]. Considering the growing demand for seaweed and seaweed-derived products, the objective of this study is to develop a green and efficient extraction method for obtaining sodium alginate from *L. japonica*. Therefore, we have developed a novel HPH pretreatment method exhibiting excellent industrial potential for producing SA.

## 2. Materials and Methods

### 2.1. Materials and Reagents

*L. japonica* was obtained from Yantai, Shandong province, China. Cellulase (3.5 × 10^4^ U/g) and pectinase (1.0 × 10^5^ U/g) were purchased from Guangxi Pangbo Biological Engineering Co., Ltd. (Nanning, Guangxi, China). Papain (6.0 × 10^5^ U/g) was provided by the Institute of Biology, Guangxi Academy of Sciences (Nanning, Guangxi, China). All chemical agent were purchased from Sangon Biotech (Shanghai) Co., Ltd. (Shanghai, China).

### 2.2. Extraction Process

1. *L. japonica* was soaked in fresh water for 4 h and washed three times with distilled water to remove impurities. The plant material was then dried and ground into a powder.
2. A solution of *L. japonica* was prepared by soaking 2.0 g of the plant material in 200 mL of pure water for 1 h. The *L. japonica* solution was then subjected to different pretreatment methods, including HPH, UAE, CE, and CE–UC.
3. After adding 30 mL of a 2% (*w*/*v*) Na_2_CO_3_ and EDTA (with or without) solution, the homogenate was incubated at 50 °C for 3 h. The mixture was then centrifuged, and the supernatant was adjusted to the desired pH using 1 M HCl.
4. Following this, 20 mL of 10% (*w*/*v*) calcium chloride was added and the mixture was allowed to stand. The resulting precipitate was then filtered and washed twice with distilled water to obtain a yellow–white gelatinous precipitate.
5. The precipitate was dissolved in 20 mL 15% (*w*/*v*) sodium chloride solution for ion exchange. The solution was then filtrated using medical gauze. Subsequently, 100 mL of anhydrous ethanol was added to induce precipitation. The resulting white flocculent precipitates were obtained through filtration.
6. The precipitates were collected and frozen at −80 °C for 12 h, followed by freeze-drying for 8 h using a vacuum freeze dryer. The dried precipitates were then crushed to obtain crude sodium alginate.

The yield of SA was calculated using the following equation:

SA yield (%) = (m_1_/m_2_) × 100 (1)

where, m_1_ is the dry weight of obtained SA, and m_2_ is the dry weight of *L. japonica*.

**Figure 1:**
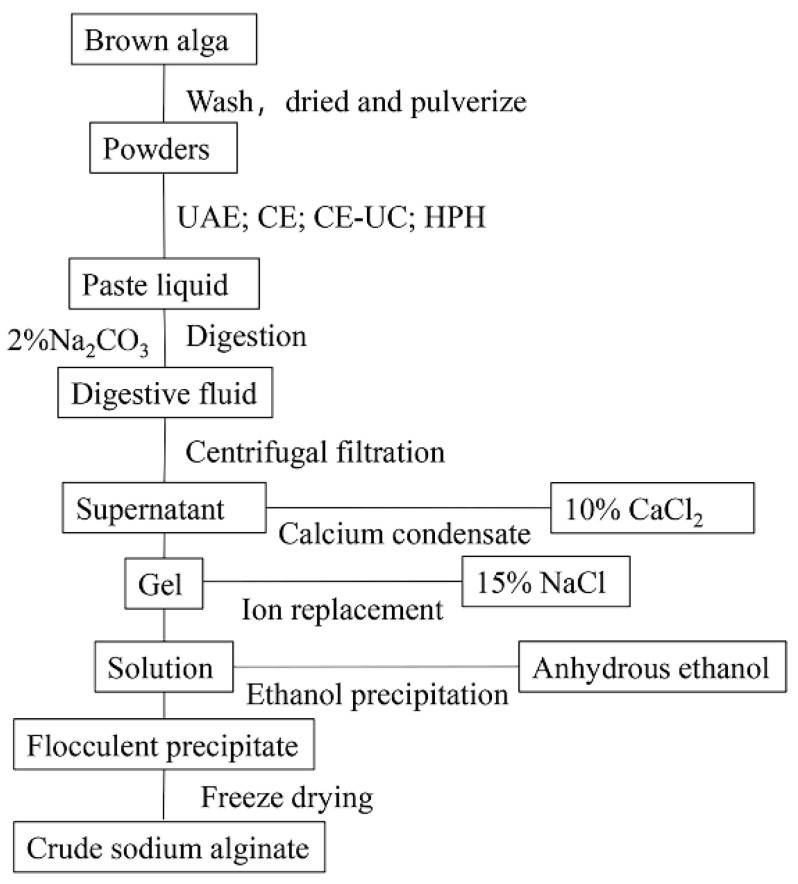
Design of the experiments for extraction.

All experiments were performed in triplicate. The standard deviations are illustrated as error bars in the figures. Above detailed steps were shown in Figure 1.

#### 2.2.1. Extraction Process of the CE Method

1. Of the *L. japonica* powder with a 100-mesh size, 2.00 g was taken and tap water was added in a 1:50 ratio to obtain a total volume of 100 mL.
2. The pH value was adjusted to 6 and 3% (*w*/*v*) cellulase of the *L. japonica* powder, 3% (*w*/*v*) pectinase, and 1% (*w*/*v*) papain were added. The mixture was stirred well and transferred to a 50 °C water bath for 3 h. After the reaction, the enzyme solution was inactivated by boiling in water for 15 min.
3. Of a 2% (*w*/*v*) sodium carbonate solution, 24 mL was added and the mixture digested in a 50 °C water bath for 3 h. The digested solution was centrifuged at 8500 r/min for 10 min and the supernatant removed. The pH of the supernatant was adjusted to 6. The subsequent operations were continued as described in Section 2.2 from step (4) to step (6).

#### 2.2.2. Extraction Process of the UAE Method

1. Of the *L. japonica* powder with a 100-mesh size, 2.00 g was taken and stirred into tap water at a material-to-liquid ratio of 1:50.
2. An ultrasonic cell crusher was used to break the samples for 10 min with the following conditions: 350 W of output power, a temperature of 30 °C, and a working time and interval of 2 s.
3. A 2% (*w*/*v*) sodium carbonate solution was added (24 mL) and digested in a water bath at 50 °C for 3 h. After digestion, the enzymolysis solution was centrifuged at 8500 r/min for 10 min. The supernatant was collected and its pH adjusted to 6. The subsequent operations were the same as in Section 2.2, from (4)–(6).

#### 2.2.3. Extraction Process of the CE–UC Method

1. Of the 100-mesh size *L. japonica* powder, 2.00 g was taken and stirred into tap water at a material-to-liquid ratio of 1:50. The samples were then subjected to ultrasonic cell crushing for 10 min using a 350 W power, 30 °C temperature, and 2 s working time and intervals.
2. The pH value was adjusted to 6, and *L. japonica* powder with 3% (*w*/*v*) cellulase, 3% (*w*/*v*) pectinase, and 1% (*w*/*v*) papain were added. The mixture was stirred well and placed in a 50 °C constant temperature water bath for enzymolysis for 3 h. After the enzymolysis reaction, the enzyme solution was inactivated by boiling and heating for 15 min.
3. Of the *L. japonica* powder in a 2% (*w*/*v*) sodium carbonate solution, 24 mL was added and digested in a 50 °C water bath for 3 h. After digestion, the enzymolysis solution was centrifuged at 8500 r/min for 10 min, and the supernatant was collected and adjusted to pH 6. Subsequent operations were the same as in Section 2.2, steps (4)–(6).

#### 2.2.4. Single-Factor Experiment of the HPH Method

In order to optimize the extraction conditions using the HPH method, various factors were investigated that have an impact on the yield of sodium alginate. These factors include pressure, cycle times, pH, EDTA, and digestion time.

Homogeneous pressure (bar): Pressures of 400, 600, 800, 1000, 1200, and 1400 bar were selected, respectively, and the yield of sodium alginate was determined while keeping the other steps unchanged.

Homogenization times: Under the aforementioned optimal conditions, extraction was conducted with homogenization performed 2, 3, 4, 5, and 6 times, respectively, while keeping the other steps unchanged. The yield of sodium alginate was determined.

pH Adjustment: Under the aforementioned optimal conditions, pH values of 5, 5.5, 6, 6.5, and 7 were utilized to adjust the extraction process. The remaining steps of the procedure were kept constant in order to determine the yield of sodium alginate.

EDTA addition amount (%): 0%, 0.25%, 0.5%, 0.75% and 1% (*w*/*v*) EDTA were added to the extraction process without changing any other steps. The yield of sodium alginate was determined under these conditions.

Digestion time (h): Under the aforementioned optimal conditions, digestion was conducted for 1 h, 1.5 h, 2 h, 2.5 h, 3 h, and 3.5 h, respectively. Extraction was carried out following all other steps, unchanged, in order to determine the yield of sodium alginate.

### 2.3. Characterization of Sodium Alginate

For the SEM analysis, the untreated and pretreated *L. japonica* power samples were examined to analyze the effect of pretreatment on their structure and morphological properties. To do this, small amounts of dried *L. japonica* samples were placed on a double-sided carbon tape adhered to aluminum stubs. These stubs were then coated with a layer of gold using sputter-plating. Finally, the samples were observed using an S-3400N electron microscope from Hitachi, Japan.

For the FTIR analysis, sodium alginate samples were analyzed using the Nicolet IS 10 Fourier Transform Infrared spectrometer (Thermo Fisher Scientific, MA, USA). The samples were scanned from 4000 to 600 cm^−1^ 32 times, at a resolution of 4 cm^−1^. This was performed to investigate any changes in the functional groups.

For the MRS analysis, the sodium alginate powder was initially spread evenly on the groove of a glass slide. The Raman spectrum (Thermo Fisher Scientific, MA, USA) in the range of 3378 cm^−1^ to 50 cm^−1^ was measured, with an exposure time of 10 s for 30 repetitions.

For the NMR analysis, the procedure was performed following the guidelines of ASTM-F2259-10 (2012) using an Agilent NMR Systems 800 MHz NMR Spectrometer (Agilent, CA, USA). Initially, the sodium alginate underwent depolymerization through acid hydrolysis. A sodium alginate solution of 100 mL with a concentration of 1 mg/mL was prepared, and the pH was adjusted to 5.6 using 1 M hydrochloric acid. The solution was then heated in a water bath at 100 °C for 1 h. Subsequently, the pH was readjusted to 3.8 using 1 M hydrochloric acid, and again, heated in a water bath at 100 °C for 30 min. The pH was then neutralized to 7.2 with 1 M sodium hydroxide, and the sample was freeze-dried overnight. The resulting freeze-dried sample was dissolved in 5 mL of 99.9% D_2_O and then freeze-dried once more. Next, 10–12 mg of the sample was dissolved in 1 mL of 99.9% D_2_O. Subsequently, 0.7 mL of the alginate solution, along with 20 μL of 0.3 M TTHA (triethylenetetraminehexaacetic acid) was added to an NMR tube. The pH of the TTHA was adjusted to 5.2 using sodium deuterium chloride (DCl) and deuteroxide (NaOD), as the TTHA served as a chelator to hinder the reaction of divalent cations with sodium alginate. For the XRD analysis, the sodium alginate samples underwent various pretreatment and were commercially recorded using an X-ray diffractometer Ultima IV (Rigaku, Tokyo, Japan). The diffraction pattern was obtained by scanning the samples at a rate of 5°/s within a range of 2θ angles of 5° to 80°. Cu radiation (40 mA, 40 kV) was used during the analysis.

TGA analysis was performed on various sodium alginate samples using a TGA Q50 Thermogravimetric Analyzer instrument (TA, DE, Waltham, MA, USA). To obtain thermograms, 10 mg of sodium alginate samples were placed in an alumina pan and was purged with nitrogen at a rate of 30 mL/min. The temperature was then increased from 20 °C to 600 °C at a heating rate of 20 °C/min.

For T-AOC analysis, the assay was based on the reduction of molybdate-IV (Mo IV) to molybdate-V (Mo V) by the extracts, and the subsequent formation of a green phosphate/Mo V complex in an acidic pH. In this study, the total antioxidant capacities (T-AOC) of different sodium alginate samples were evaluated using the ammonium molybdate method, as previously described [41]. Specifically, 0.5 mL of the sample (10 mg/mL) was dissolved in 10% double-distilled water and mixed with 5 mL of the reagent, which consisted of 28 mM sodium phosphate, 0.6 M sulfuric acid, and 4 mM ammonium molybdate. The mixture was then incubated at 95 °C for 90 min and subsequently cooled. The absorbance was measured at 695 nm against the blank. The antioxidant activity was expressed as the vitamin C equivalent antioxidant capacity (VCEAC) using a standard plot.

Statistical analysis and graphing were performed using the GraphPad software program.

## 3. Results and Discussion

### 3.1. Optimization of Single-Factor Extraction Conditions for the HPH Extraction

**Figure 2:**
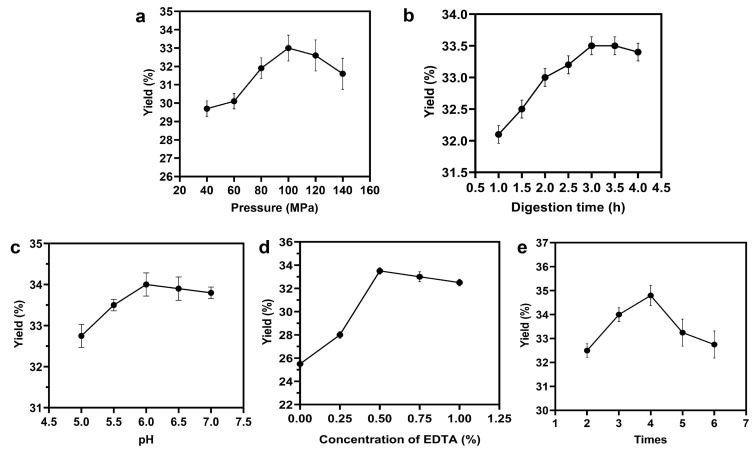
Illustration of the impact of various factors on the yield, including: (**a**) high-pressure homogenization pressure, (**b**) digestion time, (**c**) pH, (**d**) concentration of EDT, and (**e**) cycle times. A yield of 100% indicated that the weight of sodium alginate extracted was equivalent to the weight of *L. japonica*. All experiments were performed in triplicate, and data are expressed as the mean of three samples with standard deviation.

The effect of HPH pressure on yield: The effect of HPH pressure (40–140 MPa) on the yield of sodium alginate was investigated while keeping other parameters constant, including 3 cycles, pH 6.0, 0.25% (*w*/*v*) EDTA concentration, and 4.0 h digestion time. The results showed that the yield of sodium alginate increased significantly with increasing pressure from 40 to 100 MPa, but decreased after reaching 100 MPa at 1% SA concentration (Figure 2A). This observed trend is likely due to the cavitation, turbulence, and collision phenomena induced by HPH. These mechanical forces cause the material to break down and achieve super refinement, facilitating the dissolution of sodium alginate. However, at higher pressures (>100 MPa), the yield decreased as the cells are severely damaged. This may result in sodium alginate molecules being damaged into smaller sizes, which hinders the extraction process.

Effect of digestion time on the yield: In order to investigate how digestion time affects the yield of sodium alginate, the extraction process was carried out under the aforementioned optimal conditions (Figure 2B). It was observed that the yield increased as digestion time was extended, reaching its highest value (33.5%) at 3.0 h. This remarkably reduced the production cycle. However, excessively short digestion times might result in an incomplete destruction of *L. japonica*. On the other hand, if the digestion time is too long, the alginate might degrade, leading to a reduction in the yield and making the subsequent separation process more challenging. Therefore, a digestion time of 3.0 h was taken as the optimal condition.

The effect of pH on yield: The influence of pH values ranging from 5 to 7 on the yield of sodium alginate was investigated under fixed conditions of HPH pressure at 100 MPa, 3 cycles, EDTA concentration at 0.25% (*w*/*v*), and digestion time of 3.0 h. The results showed that the yield of sodium alginate initially increased and then then decreased as the pH increased (Figure 2C). Sodium alginate demonstrated excellent stability within the pH range of 6–11. When the pH was lower than 6, alginate precipitated and the yield decreased. On the other hand, when the pH exceeded 11, the alginate coagulated again.

The effect of EDTA concentration on yield: The influence of EDTA concentration on the yield of sodium alginate was evaluated under the following conditions: 100 MPa HPH pressure, 3 cycle times, pH 6.0, and 3.0 h of digestion time. The data shown in Figure 2D clearly revealed that increasing the concentration of EDTA resulted in an increase in the yield of sodium alginate, reaching a maximum of 33.5% when using a concentration of 0.5% (*w*/*v*) EDTA. This finding was significantly higher than previous reports on the extraction of sodium alginate from brown algae. The extraction process heavily relied on the complexation of calcium in the cell wall by EDTA. The optimal concentration of 0.5% (*w*/*v*) EDTA for maximizing sodium alginate yield was consistent with previous studies [42]. Additionally, including 0.5% (*w*/*v*) EDTA during the digestion stage facilitated pH adjustment to 6, reducing the amount of acid and base required for pH adjustment. However, higher concentrations of EDTA led to higher pH values, which was detrimental to the extraction process of sodium alginate. Therefore, a concentration of 0.5% (*w*/*v*) was determined to be the most effective.

The effect of HPH cycle times on yield: As depicted in Figure 2E, the yield of sodium alginate demonstrated a gradual increase with increasing cycle times. The maximum yield was achieved after 4 cycles. It should be noted that exceeding 4 cycle times would be likely to affect the degradation of sodium alginate molecules a little, due to the heating machine to a certain extent. As a result, the extraction of sodium alginate would be impeded. Hence, 4 cycle times were identified as the optimal homogenization number.

Subsequently, the optimal conditions for sodium alginate extraction were determined to be 100 MPa HPH pressure, 4 cycle times, a pH of 6.0, a 0.5% concentration (*w*/*v*) of EDTA, and a digestion time of 3.0 h. With the optimum parameters, the yield was 34%. A comparison with other reported extraction methods is necessary. The yields of different methods are shown in Table 1.

### 3.2. Compared Yield with That of Other Extraction Methods

**Figure 3:**
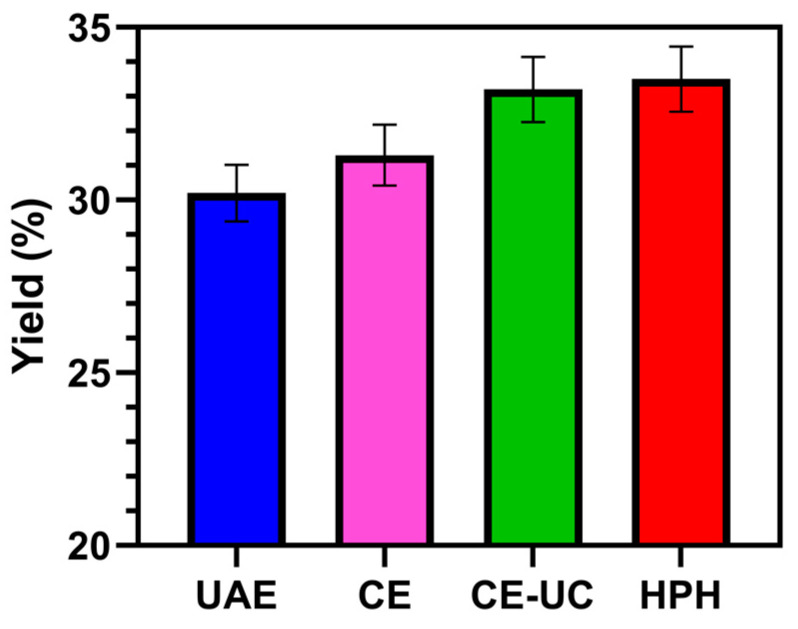
Yield of sodium alginate using different extraction methods. All experiments were performed in triplicate, and data are expressed as the mean of three samples with standard deviation.

Numerous pretreatment methods have been reported for the extraction of SA. However, most of them have certain deficiencies, such as harsh reaction conditions and high costs. In this study, we applied the HPH method to extract SA from *L. japonica* and compared it with four other methods, namely, UAE, CE, and CE–UC. The results showed that the UAE method produced the lowest yield at 30.2%, while the HPH method produced the highest yield at 34%, as shown in Figure 3. These findings demonstrated that the HPH method significantly improved the extraction yield.

### 3.3. Scanning Electron Microscopy (SEM) Analysis

The scanning electron micrographs of untreated and pretreated *L. japonica* power samples were taken at a magnification of 500× and a resolution of 100 μm. By comparing these micrographs, significant differences in surface morphology could be observed.

**Figure 4:**
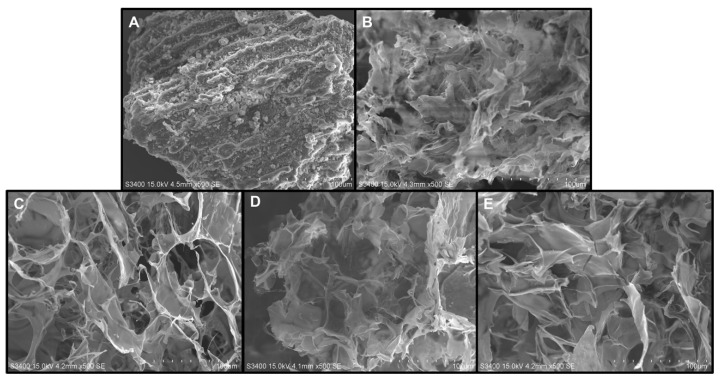
SEM micrographs of untreated and pretreated *L. japonica* power. (**A**) Untreated, (**B**) HPH, (**C**) CE-, (**D**) UAE-, and (**E**) CE–UC-pretreated *L. japonica* power at 500× magnification.

The pretreated samples appeared to noticeably change in the surface condition compared to the original sample (Figure 4). The untreated sample exhibited a tightly packed structure with intact cell walls, possibly due to the presence of strong inter- and intra-molecular hydrogen bonds in the cell wall. In contrast, the pretreated samples, including those treated with UAE, CE, CE–U, and HPH, exhibited a greater degree of surface decomposition compared to the untreated *L. japonica* powder. This observation indicated that the cell wall was significantly damaged by these pretreatment methods. Among these four methods, the sample pretreated with HPH exhibited a highly porous structure, which is likely to result in a higher extraction yield.

### 3.4. Fourier Transform Infrared (FTIR) Spectrum Analysis

**Figure 5:**
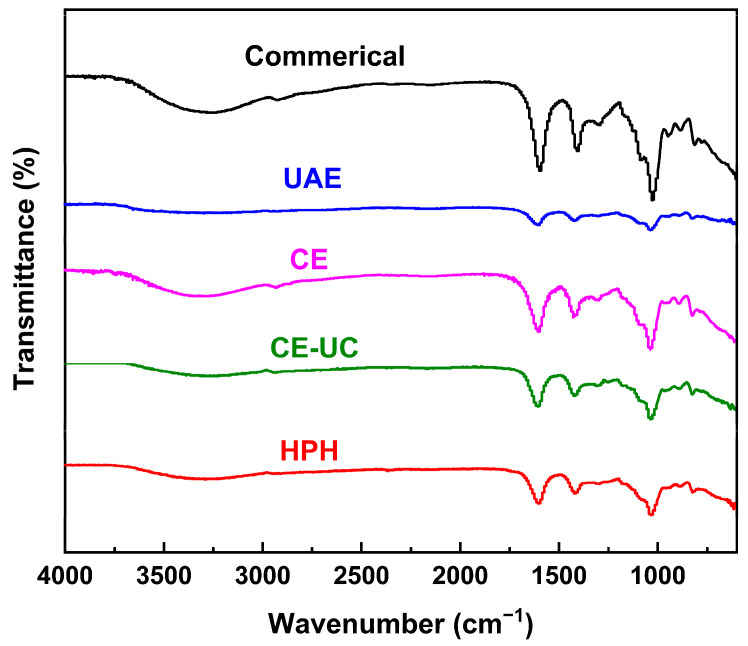
FTIR diagrams of commercial and different pretreated sodium alginate samples.

The FTIR spectroscopy results of various sodium alginate samples are shown in Figure 5, with characteristic bands consistent with those previously reported [23,45,46]. These bands confirmed the presence of the main functional groups within all samples, confirming the identity of the extracted sample as sodium alginate.

The broad band that shifted from 3400.0 to 3200.0 cm^−1^ was assigned to the O–H stretching vibration, while the band at around 2921.80 cm^−1^ was attributed to C–H stretching vibrations, including the CH, CH_2_, and CH_3_ groups. The band that shifted from 1620 to 1590 cm^−1^, as well as the band that shifted from 1420 to 1400 cm^−1^, was derived from the symmetrical stretching of the carbonyl (C=O) vibration. The band that shifted from 1200 to 1000 cm^−1^ was attributed to C–O–C and C–O–H vibrations. Additionally, the characteristic ring stretching band of a β-1,4-glycosidic bond was observed at around 886 cm^−1^. It could be observed that the intensity of the bands was reduced in the HPH method, indicating that the HPH method effectively destroyed β-1,4-glycosidic bonds and improved the yield of sodium alginate.

### 3.5. Raman Microscope Spectrometer (MRS) Analysis

**Figure 6:**
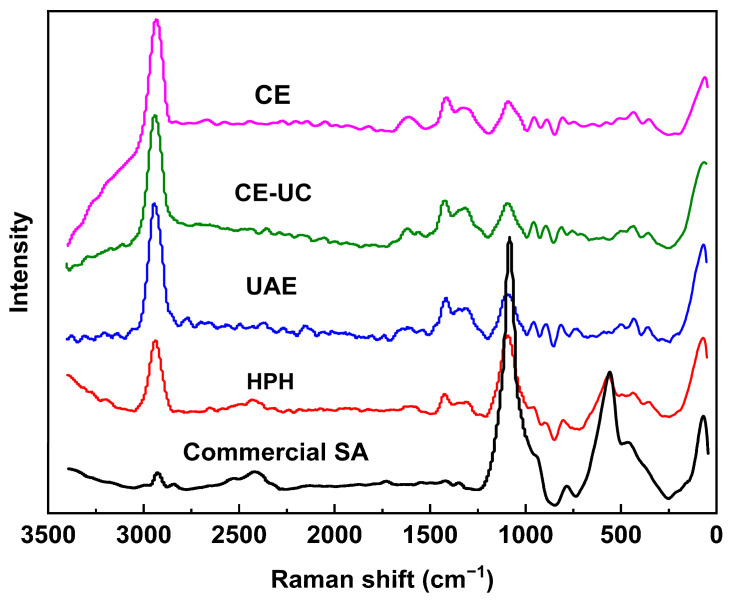
Raman spectra of different sodium alginate samples.

The characteristic peaks of SA in Figure 6 exhibited asymmetric and symmetrical stretching vibration absorption peaks corresponding to the COO– bond at about 1612 cm^−1^ and 1415 cm^−1^. The stretching vibration absorption peaks assigned to the deformed C–O–H bond, C–O bond, and C–C bond could be observed within the range of 1200–900 cm^−1^. Furthermore, the deformation vibration absorption peak of the pyranose ring and the C–O–C glycosidic bond was observed below 700 cm^−1^ [47].

### 3.6. Nuclear Magnetic Resonance (NMR) Analysis

**Figure 7:**
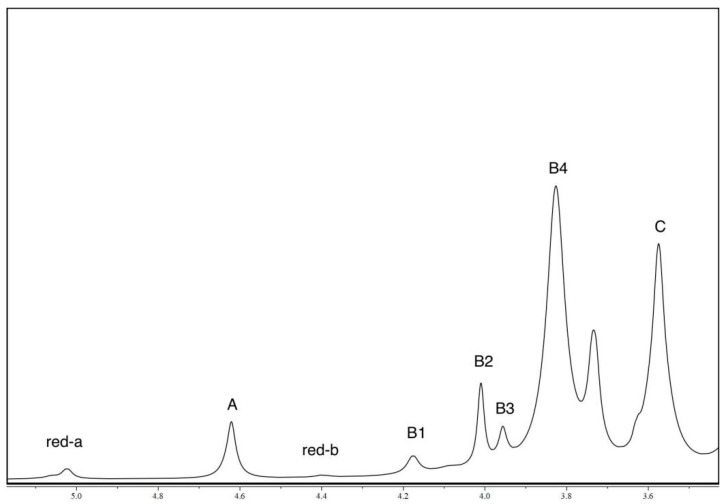
H-NMR spectra of alginate extracted by HPH. A (anomeric proton of guluronic acid), B1 (H-5 proton of the central guluronic acid residue in a GGM traid), B2 (H-5 proton of the central guluronic acid residue in a MGM traid), B3 (anomeric proton of the mannuronic acid residue neighboring a man-nuronic acid), B4 (anomeric proton of the mannuronic acid residue neighboring a guluronic acid), and C (proton 5 of guluronic acid).

Figure 7 presents the NMR spectra of the sodium alginate sample that was extracted using HPH. The peaks observed in the spectra were indicative of the presence of anomeric and other protons located at different carbon positions within the uronic acid sequence. The chemical shifts were observed at approximately 5.02, 4.62, 4.40, 4.17, 4.01, 3.96, 3.82, and 3.58 ppm, which could be attributed to A (anomeric proton of guluronic acid), B1 (H-5 proton of the central guluronic acid residue in a GGM traid), B2 (H-5 proton of the central guluronic acid residue in a MGM traid), B3 (anomeric proton of the mannuronic acid residue neighboring a mannuronic acid), B4 (anomeric proton of the mannuronic acid residue neighboring a guluronic acid), and C (proton 5 of guluronic acid), respectively. It is important to note that these chemical shifts differed slightly from those observed in the alginate extracted using the ASTM standard. This variation could be attributed to the presence of trace amounts of metal ions in the extracted alginate and the differences in its chemical composition, properties, and molecular weight. The presence of metal ions, such as calcium, could lead to a broadening of the signal lines and selective loss of signal intensity.

The block structure and M/G ratio were calculated following the calculation method outlined in the ASTM standard F2259-10 (2012). Table 2 displayed the calculated values for F_G_, F_M_, F_GG_, F_MM_, F_MG_, F_GM,_ F_GGG_, F_MGM_, F_GGM_, F_MGG_, N_G_, N_M_, DP_n_, and M/G ratio. The M/G ratio of alginate extracted from HPH was found to be 2.03, which fell within the range of the M/G ratios observed in commercially available alginate extracted from *Laminaria* (1.2–2.2) [48].

The physicochemical properties of alginate were greatly influenced by its structure, including factors such as the M/G ratio and the arrangement of the M and G residues [49]. The uronic acid sequence played a crucial role in determining the gel-forming ability of alginate. Alginates with a high M/G ratio tended to form gels that were soft and elastic [50]. Therefore, as the extracted alginate in this study had a high mannuronic acid content of 67%, it was likely to form soft and elastic gels.

### 3.7. X-ray Diffraction (XRD) Analysis

**Figure 8:**
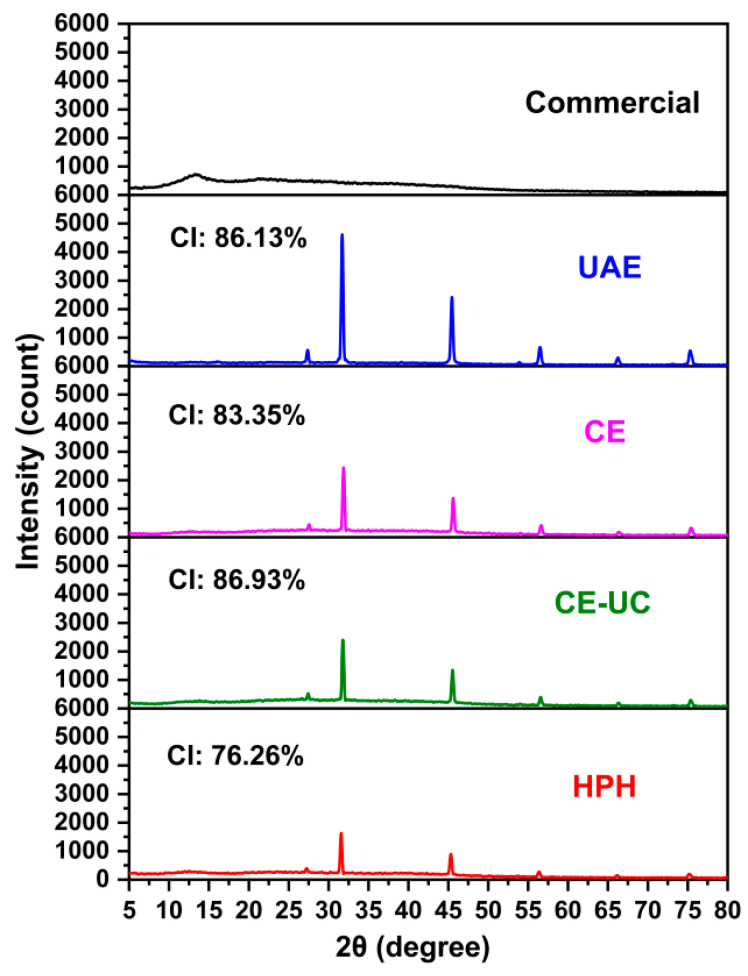
X-ray diffraction spectra of different sodium alginate samples.

XRD analysis was conducted on various sodium alginate samples to investigate their crystallinity and recalcitrance properties. The crystallinity of sodium alginate was a key attribute in determining its efficiency for enzymatic hydrolysis. A comparative X-ray diffractogram of the different sodium alginate samples was shown in Figure 8. The HPH method resulted in a crystallinity of 76.26%, which was the lowest among all the samples, excluding commercial sodium alginate. The diffraction pattern of commercial sodium alginate only showed a peak at around 13.3° and a very weak diffraction peak at 22.5° [51]. The other samples exhibited characteristic strong peaks at 2θ values of 31.8°, 45.4°, 56.6°, and 66.2° [52]. Notably, the sodium alginate extracted using the HPH method displayed decreased intensities of characteristic peaks, suggesting that the HPH method induced amorphousness in sodium alginate, enhancing the accessibility of alginate lyase, and promoting the production of alginate oligosaccharides.

### 3.8. Thermal Gravimetric Analysis (TGA) Analysis

**Figure 9:**
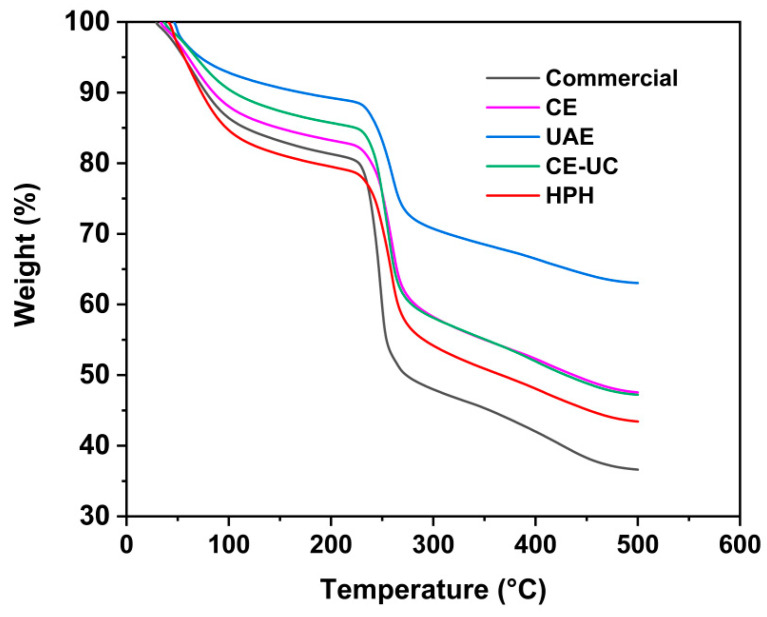
TGA thermograms of different sodium alginate samples.

Thermal gravimetric analysis (TGA) was performed to investigate the thermal stability of various sodium alginate samples. The TGA curve for sodium alginate displayed three distinct weight loss steps during thermal decomposition (Figure 9). In contrast to the commercial sodium alginate, TGA curves of the extracted samples exhibited a tendency toward higher temperatures, indicating an enhanced thermal stability. The removal of water molecules from sodium alginate occurred within the temperature range of room temperature to 120 °C. During this interval, the sodium alginate extracted using the HPH method exhibited the highest weight loss (17%), while the sodium alginate extracted using the ultrasonic method underwent the lowest weight loss (8%).

After the loss of water, sodium alginate decomposed at temperatures ranging from 210 °C to 300 °C. For commercial samples, decomposition occurred at 220 °C, with completion at 260 °C. Sodium alginate extracted through HPH degraded within a temperature range of 240 °C to 280 °C. The result of TGA displayed a strong correlation with the result of XRD, indicating that the reduction in crystallinity facilitated the degradation of the HPH-extracted sample. These findings were consistent with previous studies [53].

### 3.9. Total Antioxidant Capacity Assay (T-AOC)

**Figure 10:**
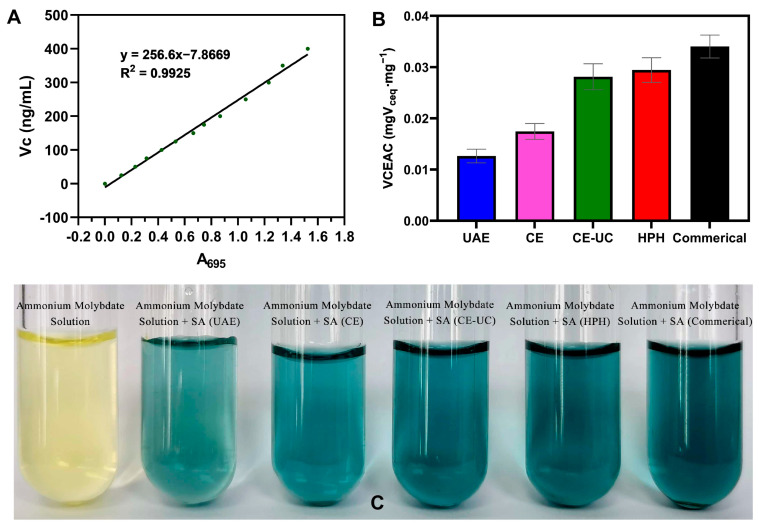
Standard curve of (**A**) V_C_ and (**B**,**C**) V_C_ equivalent of the five sodium alginate samples.

The results of the antioxidant capacity of various extracted sodium alginate samples are presented in Figure 10, and their antioxidant activities were evaluated as vitamin C equivalent antioxidant capacity (VCEAC). Among these samples, sodium alginate extracted using UAE showed the lowest antioxidant capacity (0.01264 mgVceq∙mg^−1^), whereas the sodium alginate extracted using HPH displayed the strongest antioxidant capacity (0.02942 mgVceq∙mg^−1^), second only to commercially available sodium alginate. These findings could be partially explained by the different molecular weights of sodium alginate extracted using different methods. It is well-known that polysaccharides with lower molecular weights exhibit better antioxidant activity.

## 4. Conclusions

In this paper, we investigated the successful application of calcium coagulation ion exchange combined with high-pressure homogenization for extracting sodium alginate from brown seaweed. We found that HPH effectively broke the cell wall of the *L. japonica*, resulting in an improved yield, faster production, and reduced costs. Moreover, the sodium alginate extracted using this method exhibited stronger antioxidant activity. These findings present a promising new strategy for the industrial extraction of sodium alginate.

Furthermore, alginate oligosaccharides, which were the degradation products of alginate, have attracted considerable attention owing to their notable physiological characteristics. These include immune regulation, antimicrobial properties, antioxidant activity, antitumor effects, anti-inflammatory properties, and the promotion of plant growth. The low crystallinity of sodium alginate extracted in this work was advantageous for the production of AOS and effectively supported the advancement of their industrial production.

## Figures and Tables

**Table 1:** Sodium alginate yields for crude extracts compared to previous literature.

Species	Method	Optimal Extraction Conditions	Yield (%)	Reference
*Laminaria* *japonica*	HPH method	100 MPa HPH pressure, 4 cycle times, a pH of 6.0, a 0.5% concentration (*w*/*v*) of EDTA, and a digestion time of 3.0 h.	34%	This study
*Laminaria* *japonica*	CE method	3% cellulase of *L. japonica* powder, 3% pectinase, and 1% papain.	31.3%	This study
*Laminaria* *japonica*	UAE method	350 W power, 30 °C temperature, and 2 s working time and intervals.	30.2%	This study
*Laminaria* *japonica*	CE–UC method	3% cellulase, 3% pectinase, and 1% papain; and 350 W power, 30 °C temperature, and 2 s working time and intervals.	33%	This study
*Laminaria* *japonica*	Ultrasonic complex enzymatic hydrolysis method	Cellulase concentration 0.3 g, pectinase concentration 0.3 g, papain concentration 0.1 g, enzymolysis pH = 4, enzymolysis temperature 55 °C, ultrasonic power 250 W.	21.53 ± 0.12%	[29]
*Laminaria* *japonica*	Enzyme–Ultrasonic combined method	Enzyme (cellulose and protease add content) 8% (*w*/*v*), pH 4, temperature 50 °C, ultrasonic power 160 W, enzyme solution time 3.5 h.	23.1%	[30]
*Nizimuddinia zanardini*	Microwave-assisted extraction	Temperature of 67 °C, microwave power of 400 W, and solvent/biomass ratio of 29 mL/g after 19 min.	31.39%	[43]
*Sargassum*	Alkaline extraction	12.63 mL of 3.75% (*w*/*v*) Na_2_CO_3_ for 6 h at 80 °C	20.76 ± 0.73%	[44]

**Table 2:** Uronic acid sequence and the M/G ratio of sodium alginate extracted by HPH.

F_G_	F_M_	F_GG_	F_MM_	F_GM_ = F_MG_	F_GGG_	F_MGM_	F_GGM_ = F_MGG_	M/G Ratio	N_G_	N_M_	DP_n_
0.33	0.67	0.13	0.47	0.20	0.05	0.11	0.09	2.03	1.65	3.35	14.72

## Data Availability

Not applicable.

## Abbreviations

HPH: high-pressure homogenization method; MAE: microwave-assisted extraction; UAE: ultrasonic-assisted extraction; CE: complex enzyme hydrolysis; CE–UC: complex enzyme–ultrasonic combined method; SEM: scanning electron microscopic; FTIR: Fourier transform infrared; MRS: microscope Raman spectrometer; NMR: nuclear magnetic resonance; XRD: X-ray diffraction; TGA: thermal gravimetric analysis; T-AOC: total antioxidant capacity assay.
